# Effect of low-fusing porcelain glaze as zirconia surface treatment on the adhesion of resin cement

**DOI:** 10.4317/jced.62601

**Published:** 2025-04-01

**Authors:** Hasan Skienhe, Ahmed Abdou, Jukka P. Matinlinna, Marco Ferrari, Ziad Salameh

**Affiliations:** 1 Department of Prosthodontics, Faculty of Dental Medicine, Lebanese University, Lebanon; 2 Department of Restorative Dentistry, Faculty of Dentistry, Universiti Malaya, Kuala Lumpur, Malaysia; 3 Applied Dental Sciences, Biomaterials Science, Division of Dentistry, The University of Manchester, Manchester, M13 9PL, United Kingdom; 4 Department of Prosthodontics, Faculty of Dental Medicine, University of Siena, Italy

## Abstract

**Background:**

This laboratory study aimed to investigate the influence of a novel surface treatment method, utilizing a low-fusing porcelain glaze layer, on pre-sintered zirconia and the adhesion with resin composite cement. The study employed a primer containing a silane coupling agent and 10-methacryloyloxydecyl dihydrogen phosphate (MDP) to enhance the bond between the zirconia and resin composite cement.

**Material and Methods:**

Zirconia blocks were sectioned, polished, and randomly divided into six groups based on surface treatment before sintering: Group CA50: Pre-sintered specimens were grit-blasted with 50 µm Al2O3 particles; Group CS25: Pre-sintered specimens were grit-blasted with 25 µm high-purity fused silica particles; Group B: Specimens similar to Group CA50 were sintered, glazed, and fired, followed by etching with 9.5% hydrofluoric acid (HF); Group C: Specimens similar to Group CA50 were sintered, glazed, and fired, followed by re-blasting with grit-blasted with 50 µm Al2O3 particles, Group D: Specimens similar to Group CS25 were sintered, glazed, and fired, followed by HF, Group E: Specimens in Group CS25 were sintered, glazed, and fired, followed by sandblasting with 50 µm Al2O3 particles. Microstructural analysis, surface roughness analysis, and shear bond strength testing (SBS) were conducted.

**Results:**

The surface treatment significantly increased the surface roughness with all surface treatments compared to CA50 and CS25. The shear bond strength of group C showed the highest significant SBS compared to all other groups.

**Conclusions:**

Alumina sandblasting of the glazed zirconia as pretreatment of sintered zirconia can improve the bonding to silane primer and resin cement.

** Key words:**Glass beads, Alumina blasting, Pre-sintered zirconia, Silane.

## Introduction

The yttria-stabilized tetragonal zirconia polycrystal (Y-TZP) type represents one of the reinforced ceramic materials utilized in the CAD/CAM technology. It is used as a core material in fixed dental restoration as it has excellent biocompatibility, fracture toughness, aesthetics, and strength (1,2). Nevertheless, it is challenging to produce a reliable and durable chemical bond between resin composite cement and zirconia due to the zirconia composition which is free from etchable silica (SiO2) and makes zirconia an acid-resistant substance (3). Surface roughening from etching improves the adhesion between the cement and ceramic which relies on micro-mechanical interlocking (4).

Micro-mechanical interlocking to silica-based ceramic obtained by hydrofluoric acid (HF) etching by which dissolving the glassy matrix increases the surface roughness (Ra) and the total surface area for bonding (5,6). This procedure is generally followed by a silane coupling agent the application (which is a synthetic hybrid Si-C monomer) that promotes adhesion between organic and inorganic counterparts (7). As silane will increase the surface free energy (lowering surface tension) and wettability of ceramic surfaces, both of which enhance the chemical adhesion (8).

One of the key elements of a zirconia restoration’s clinical effectiveness is adhesive cementation (9). It improves the retention and marginal adaptation of restoration, as well as, improving the strength, absorbs biting forces, and reduces the possibility of recurrent decay. Changing surface topography (roughening) using various methods has been applied to promote mechanical adhesion between zirconia and resin cement (10). The most common method is grit-blasting using aluminum trioxide (Al2O3) (11). However, studies have shown low bond strength values with grit-blasting or even spontaneous debonding after artificial aging (12,13). Moreover, a decrease in fracture strength of zirconia as a result of eventual surface damage and micro-crack formation after grit-blasting (14-16). Even so the tetragonal-to-monoclinic phase transformation was increased after grit-blasting (17,18).

There is a wide understanding that the success of adhesive cement with a zirconia restoration is directly related to chemical bonding and micro-mechanical interlocking between the cement and the inner surface of the restoration (19). Different surface treatment methods have been developed to utilize the chemical bonding to zirconia ceramics including( different coating technique) internal coating technique (20), nanostructured alumina coating (21), zirconia powder coating, and silica coating methods (22-26). Given this, the application of the coating on the intaglio surface of zirconia restoration may increase the marginal misfit of the zirconia restorations (56.5 μm to 215.0 μm.) (27).

Grit-lasting of zirconia before sintering has appeared to be a useful surface treatment method to increase the surface area and roughness. As well the monoclinic phase transformation was almost zero in sandblasted zirconia after sintering (28,29). However, no current studies found in the literature have evaluated the deposition of silica layer on sintered zirconia after being grit-blasted by different abrasive materials before sintering.

The purpose of this study was to investigate the effect of a novel zirconia surface treatment method using a low-fusing porcelain glaze layer as an experimental procedure for promoting the bond strength between zirconia when MDP and silane-containing resin cement was employed. Moreover, to estimate the glaze layer thickness after being treated with alumina grit-blasting and HF acid. The null hypothesis to be tested was that (a) there will be no significant increase in the bond strength between zirconia and resin cement, and (b) the thickness of the glaze layer after surface treatment will not be within the acceptable clinical range.

## Material and Methods

The materials used in this laboratory study are shown in  Table 1.

-Specimen’s preparation

A non-sintered (Y-TZP) 3 mol% yttria-stabilized zirconia block (Amann Girrbach, Koblach, Austria) was used. One hundred twenty square-shaped specimens (3 mm × 12 mm × 12 mm ) were sectioned out of the zirconia block using a low-speed diamond disc (Buehler, Lake Buf, Wisconsin, USA). Then, samples were polished with silicon carbide grit papers (Gritfex, Italy) #800, 1000, 1200, 1500, and 2000 to ensure identical initial roughness. A pilot study was done before the current study with a large effect size of 2.25, a power of %82, and a significance level of 0.05 for sample size determination.

-Surface treatment protocols 

Specimens were randomly divided into 6 groups (n=20 each,) according to the surface treatment into:

1. Group CA50 (Control 1): the surface of the pre-sintered specimen was grit-blasted with 50 μm Al2O3 particles for 7 s under 2 bar pressure. The nozzle was placed perpendicularly to the specimen surface (3 cm away from the surface). Then, Specimens were sintered according to the manufacturer’s instructions.

2. Group CS25 (Control 2): the surface of the pre-sintered specimen was grit-blasted with 25 μm high fused silica particles for 7 s under 2 bar pressure. The nozzle was placed 3 cm away and perpendicular to the specimen surface. The nozzle was placed perpendicularly to the specimen surface (3 cm away from the surface). Then, Specimens were sintered according to the manufacturer’s instructions.

3. Group B: Same as group CA50. Then, a thin film of glaze was applied to the zirconia surface. Subsequently, 9.5 % of hydrofluoric acid (HF) was applied on the zirconia surface for etching for 25 min and rinsed with water.

4. Group C: same treatment as group B, but HF application was replaced with grit-blasting with 50 μm Al2O3 particles under 3 bar air pressure of for 10 seconds after the glaze film application. The nozzle was placed 15 mm away and perpendicular to the specimen surface.

5. Group D: the surface of pre-sintered specimens was grit-blasted with 25 μm high fused silica particles for 7 s and under 2 bar pressure. The nozzle was placed 3 cm away and perpendicular to the specimen surface. Then, the specimens were then sintered according to the manufacturer’s instructions. Then, a thin glaze film (Ivoclar Vivadent, Schaan, Liechtenstein) was applied to the zirconia surface. After that, 9.6 % of hydrofluoric acid was applied to the zirconia surface for etching for 25 min and rinsed with water.

6. Group E: same treatment as group D. The glazed surface was grit-blasted with 50 μm Al2O3 powder for 10 s under 3 bar air pressure after sintering instead of HF application in group D. The nozzle was placed 15 mm away and perpendicular to the specimen surface.

-Glaze layer application

A thin film of ceramic glaze was applied using a fine ceramic brush and allowed to dry at 400 C for 6 min. The samples were heated up to 830 ˚C, 50 ˚C per minute under vacuum which began at 450 ˚C, and was held for 1 min at 830 ˚C using (Programat P300, Ivoclar Vivadent, Schaan, Liechtenstein) and then cooled at room temperature.

The HF acid etching time and grit-blasting protocol were based on a pilot evaluation. The HF acid etching time that was used was 25 min since at this time the zirconia surface appeared through Scanning electron microscopy with some traces of glaze. The same was carried out for the grit-blasting protocol that was observed for different pressures.

-Microstructural analysis

Scanning electron microscopy (SEM) and Energy Dispersive Spectroscopy (EDX)

Two specimens from each group were gold-sputtered (Sputter Coater 108 Auto, Cressington Scientific Instruments, Watford, UK) and examined using an SEM device (IS2100C, Seron Technologies, ASI2100, Gyeonggi-Do, Korea) at 1000× magnification and 20 kV. Another 2 specimens were also selected for surface elemental analysis (EDX analysis, AMETEK with an EDAX detector). Two more specimens from each of the groups B, D, and E were prepared and surface treated as described in the surface treatment protocol. Next, the specimens were bonded to polished zirconia slabs using a primer and resin composite cement. Subsequently, the bonded specimens were cut straight in the middle and polished, then the specimens were gold sputtered and examined by SEM for the glaze thickness estimation and resin composite cement.

-Profilometer

A total of 18 specimens (n=3, for each group) were used for the surface roughness (Ra) analysis using a profilometer (Daily aid DR 300, Beijing, China). Three areas on each specimen were randomly selected and analyzed, each sample was measured 3 times at different random locations with a moving distance of 2.5 mm across the zirconia surface. The mean used for Ra calculation.

Shear bond strength testing (SBS)

One hundred twenty resin composite cylinders (Z 250, 3M ESPE, Saint Paul, MN, USA), of 4 mm diameter and 4 mm high were prepared using a Plexiglas mold. The resin composite was light-cured using a halogen light source (Elipar FreeLight 2 LED, 3M ESPE) for 40 s on the top and then 40 on the bottom surface of the resin composite cylinders. The adhesive system that was used is dual-cured resin cement (RelyX Ultimate, 3M ESPE) with primer (Scotchbond Universal, 3M ESPE), which contains a phosphate ester monomer MDP and silane. Specimens were mounted in auto-polymerizing acrylic resin, using a custom-made mold to obtain a smooth flat surface. A thin layer of primer was applied for 20 s on the zirconia specimens using a fine micro-brush. The resin composite cylinders (4 mm diameter and 4 mm height) were cemented on the zirconia surface using a duel-cured resin cement and a fixed load of 500 g was applied for 50 s. The excess cement was removed using a fine micro-brush. Light curing at the interface area for 40 seconds from 3 different directions. Specimens were stored in distilled water for 24 h at 37ºC and then shear bond strength (SBS) was tested using a universal testing machine (YL-UTM Main, YLE GmBH, Bad Konig, Germany). A semi-circle uni-bevel chisel-shaped indenter was used to direct the shearing force at the zirconia composite interface with a crosshead speed of 1.0 mm/min until failure. The load was recorded in Newtons and converted to MPa by dividing it by the measured surface area. All failed samples were analyzed using SEM to evaluate the mode of failure. Failure may take place cohesively within the cement or composite and adhesively between cement and zirconia. Mixed failure shows a partly covered zirconia surface by the remaining resin cement or resin composite.

-Statistical analysis

The data were collected for statistical analysis using a statistical software package (SPSS version 23, Armonk, NY, USA). Shapiro-Wilks tests done to the six groups revealed that the groups were normally distributed. Accordingly, one-way analysis of variance (ANOVA) was done to detect statistical significance among the groups. The level of statistical significance was set at an alpha level of 0.05. Post hoc comparisons using the Tukey HSD test were performed.

## Results

-SBS results

**Figure 1:**
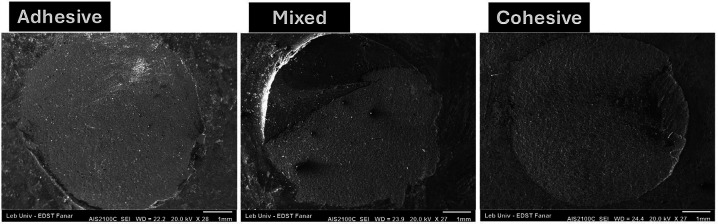
Representative SEM image for failure mode.

 Table 2 shows the SBS values of the studied groups. The ANOVA test was very highly statistically significant F(5,78)=17.92, *p*<0.001. The highest significant SBS value was for group C compared to all tested groups. The lowest significant SBS value was for the CS25 compared to all groups except CA50. There was no significant difference between the groups B, D, and E. The failure mode of groups B, D, and E were mixed whereas for group C it was both cohesive and mixed. Representative images for failure mode are presented in Figure 1.

surface roughness (Ra) 

The results of surface roughness are presented in  Table 2. CS25 group showed a significant decrease in Ra compared to CA50. All surface treatments (Group B, C, d, and E) showed a significant increase in the surface roughness compared to both CA50 and CS25 ( *p*<0.05) with an insignificant difference between each other’s.

-SEM/EDX results

**Figure 2:**
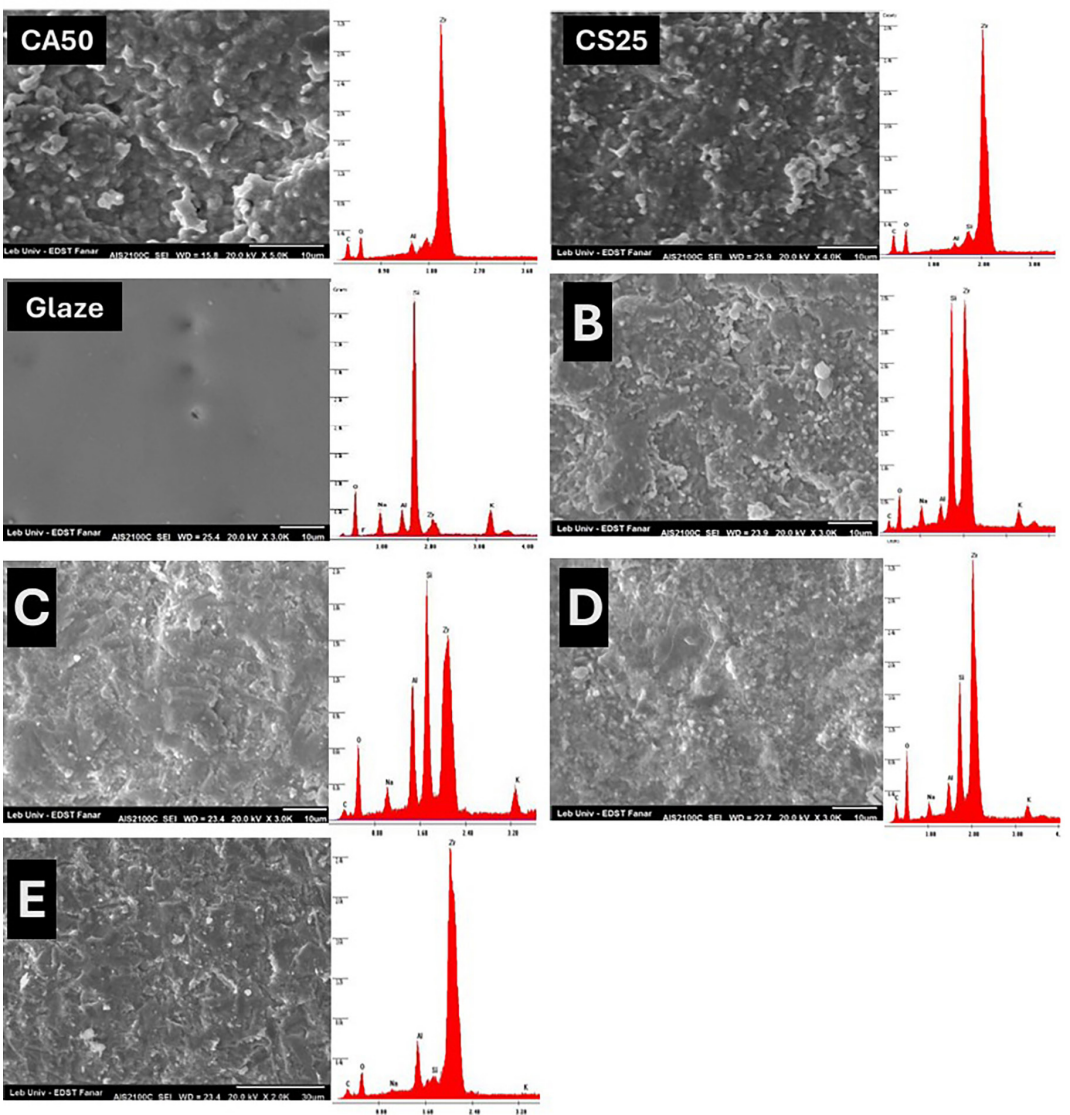
SEM/EDX of the tested groups. B,C,D, and E groups showed irregular surfaces as a result of Al2O3 sandblasting and hydrofluoric acid etching to the glazed surface, however, the Si content for groups B and C was higher compared to groups D and E.

SEM and EDX images are presented in Figure 2. CA50 showed an increase in the surface irregularities compared to CA25. However, both should profound zirconia peak on the surface with a very small presence of Si on the CS25. Glazed samples showed complete coverage with only Si presented on the surface. For group B, the surface for irregular with the presence of Si and Zr on the surface. For group C, the rough surface with Al showed a higher percentage with Si and Zr. For group D, samples showed an irregular rough surface with high Zr % with lower % for Si and Al. For group E, the Si% on the surface is low compared to Al and Zr. The thickness of the glazing layer after sandblasting or HF etching is illustrated in Figure 3. The thickness of the glaze layer (75.49 µm). The glaze thickness for group B (after HF application, 49.71 µm) was thicker than that of group C (after sandblasting, 37.3 µm).

## Discussion

The null hypothesis regarding the shear bond strength between zirconia surface and resin cement was rejected since the results of all groups showed a highly significant increase in the SBS value when compared to both control groups.

Durable adhesion to zirconia restoration relies on micro-mechanical interlocking (retention) deriving from surface roughening between the ceramic surface and adhesive resin composite cement (30).

Chemical bonding by HF acid etching comes to question when it is used as a recommended method to pretreat (roughen) silica-based ceramic surface (31), or by chemical bonding between 10-MDP and zirconia. Nagaoka N *et al*. (32) stated that the bond between 10-MDP and zirconia was not only ionic bonding but also based on hydrogen bonding. They could confirm that the 10-MDP monomer can be adsorbed onto the zirconia particles via hydrogen bonding or ionic interaction between the P-OH and Zr-OH groups or between P-O− and partially positive Zr ions, respectively. However, the inherent lack of silica in zirconia eliminates the possibility of application of chemical bonding between silica-based surfaces and a silane coupling agent and resin composite cement. Application of silica to the surface of zirconia using glass beads and tribochemical coating showed promising results; however, concerns of the separation of the silica layer from the zirconia surface may affect the longevity of the restoration (33,34).

That said, the coating of a zirconia surface by silica after being grit-blasted by alumina powder has been an attempt to increase the embedded silica content that could be dissolved with hydrofluoric acid to create pits and grooves. This way, the silane that is present in a primer could form siloxane bonds (-Si-O-Si-O-Si-) with the silica, along with the chemical bond that could be achieved by using MDP.

By and large, the surface treatment methods are either subtractive or additive (35). In the current study, the two treatments applied were grit-blasting before sintering followed by an application of low fused silica after sintering. The results of the current study suggest that group C exhibited the highest and significantly different SBS value. This was even though it had the same Ra value as the other study groups. This supposedly reveals that the factors needed for the bond to zirconia ceramic were achieved in this group due to a combination of mechanical retention (by grit-blasting) and chemical reaction by the primer containing 10-MDP (36) and silane (7).

The SEM images of the failed samples in group C showed and suggested a mixed and cohesive failure mode within the resin composite cylinders and resin composite cement. Even so, in group E which was grit-blasted with the same protocol as group C, the failure mode was mixed and adhesive - and the SBS value was significantly lower than that of group C.

The highest significant Ra value of group CA50 before the application of glaze could aid in retaining at least some silica traces in the grooves that were created after pre-sintering air abrasion. This was confirmed by SEM and EDX of groups C and B after grit blasting and HF etching. The higher, significantly different SBS value for group C could be related to its higher Ra value before the glaze application when compared to the Ra value of group E. It should be noted that both groups received the same grit-blasting protocol after the glaze application. Grit-blasting of the glazed layer of group E could have left a smaller amount of silica on the surface compared to group C, due to the lower Ra value of group E before the glaze application. This also was confirmed by SEM and EDX of groups C and E after grit blasting. Consequently, the silane present in the primer could not work properly in group E as in group C. This might explain the higher SBS value of group C. As a result, we might state that the amount of silica retained on the surface of group C could be more than that retained in group E, and potentially, a chemical reaction could be achieved in this group by 10-MDP and silane. The same supposedly occurred in group E. It showed a highly significant SBS value compared to CS25, this could be due to the significant difference in surface roughness value before glaze coating and after grit blasting and the presence of silica on the zirconia surface.

These results in accordance with previous studies (7,32,37-39) reliably showed that the adhesion and durable bond to zirconia surface relies on the combination of mechanical interlocking and chemical bonding.

The other groups that were treated by HF acid etching (B and D) created significantly higher SBS values than that of group CS25, and lower SBS values than that of group C despite having very close Ra values with no significant difference after treatment of the glazed layer. Inherently, all the groups have a glazed layer thickness that ranges between 50 μm and 60 μm. Since the etching time was the same for all the samples, still some of them may have been covered by glaze preventing the adhesive monomer 10-MDP from being in direct contact with the unexposed zirconia surface. This said the bonding of 10-MDP to the glazing material used should be studied in detail in the near future. Even so, this might explain the difference in SBS values for groups B and D vs. group C.

Comparing and contrasting the groups B and D to CA50 shows no significant difference in SBS values. The bonding mechanism appears more chemical between silane and silica than micromechanical. This could be explained by SEM of groups B and D which shows a surface lost a part of its roughness due to the retained silica traces in the grooves, in addition to the areas that are covered by glaze. However, those groups show a highly significant difference compared to CS25, So the reaction between 10-MDP and exposed zirconia could be weak and needs further investigation. Nevertheless, the failure modes of those samples were mixed and adhesive.

Cura *et al*. (5) reported that the use of silane on glazed zirconia etched by HF acid significantly increased the SBS of resin composite cement to zirconia. However, the SBS was not increased significantly when a primer containing 10-MDP was used instead of silane. It is noTable that, 60 s HF acid etching time, created a roughness that is still on the surface of the glaze layer, and the adhesive monomer 10-MDP used did not work.

The results of the current study following many studies (25,37,40,41) that stated the possibility of micro-mechanical bonding based on HF acid etching, and a chemical bonding between silane and deposited silica via siloxane bond formation. On the other hand, these results were not in agreement with those of Ashkan Moradabadi *et al*., (42) which concluded that micro-mechanical retention is a more effective mechanism than chemical adhesion to obtain higher bond strengths between zirconia and resin composite cement. The latter depended on the contact mechanics theories (43,44) that stated that an increase in the bond strength is caused by the increase in surface roughness, which in turn can be attributed to a micro-mechanical retention mechanism.

Also, the results of Valentino *et al*. (45) and Vanderlei *et al*. (46) were not in agreement with the current ones, which stated that the bond based on grit-blasting was lower than by etching.

Many previous studies (45,47,48) used a short etching time on coated zirconia surfaces to create a rough surface for mechanical interlocking (retention). Adhesion persists within the glaze layer since the 60 s to 180 s of etching time that was used was not able to dissolve a large part of this silica-rich layer. Theoretically, this might affect the marginal integrity of the restoration when we estimate the thickness of glaze and resin composite cement. Given this, the fitting surface of the restoration should be considered in any surface treatment, and efforts were made in the current study to create a minimum glaze and cement thickness. The SEM analysis shows a variation in the glaze layer thickness ranging between 45 μm and 60 μm, which could be explained by the fact that applying the glaze layer with a fine brush on the surface of a large number of samples could not be controlled even with an experienced dental technician.

Till now, the literature has not defined the minimum clinically acceptable marginal fit of CAD-CAM zirconia restoration. This said Abduo *et al*. stated (27) that the internal fit of zirconia restorations reported in different studies ranged from 56.5 μm to 215.0 μm. Even so, many studies (49-51) agree that it should be less than 120 μm. Others (52,53) consider that the marginal discrepancy should not be more than 100 μm in the era of CAD-CAM technology.

In the current study, the SEM analysis shows that the thickness of glaze and cement ranges between 30 μm and 50 μm, for alumina grit-blasted and hydrofluoric acid groups. In contrast, it shows a value of 75 μm for the non-treated glaze layer, this reveals that the cement (the ultimate resin cement) thickness was approximately 12 μm, within the rated range stated by the manufacturer (12 μm). The glaze and cement thickness after treatment was greater in the groups treated with HF acid compared to the groups treated by grit-blasting.

Grit-blasting with 50 μm Al2O3 of the glaze layer applied after sintering on the zirconia surface treated in the pre-sintered stage suggests that some important factors needed for the optimum bond to zirconia ceramic were achieved. Besides, the minimal glaze-cement thickness was achieved in this study group.

Since cement and glaze thickness in the current laboratory study are still within the minimum acceptable range reported in the literature, and the adhesion strength is higher than the clinically acceptable range, the evaluation of the coated treated zirconia should be applied experimentally on the intaglio surface of zirconia crown. Given that the thickness created was done on a zirconia slab rather than on a real zirconia crown, it presents a limitation of the testing methodology. Moreover, thermocycling could further indicate the longevity of the bonds with other clinical scenarios for validation of the suggested pretreatment. The zirconia treatment was applied on pre-sintered zirconia, which is not currently the gold standard for zirconia pretreatment. The results of alumina blasting before and after sintering showed similar outcomes (38). Alumina blasting was chosen before pre-sintering in CA50 as a control due to its similarity with other groups.

## Conclusions

Glazing zirconia before sintering resulted in improved bonding to zirconia particularly with silane and 10-MDP primer. Grit-blasting with 50 μm Al2O3 after sintering resulted in higher bond strength to zirconia. HF acid etching of the glaze layer applied after sintering might be a valuable surface treatment method.

## Figures and Tables

**Table 1:** Materials used and their manufacturers.

Material	Composition	Lot/Manufacturer
Zirconia: Ceramil Zi	Yttria stabilized polycrystalline tetragonal zirconia (Y-TZP) blank (ZrO_2_ + HfO_2_ + Y_2_O_3_ 99.0)	1512006-1/Amann Girbach
Primer: Scotch bond Universal	10-MDP, Vitrebond copolymer, HEMA, silane, dimethacrylate resins, fillers, initiators, ethanol	635860/3M ESPE,
Resin composite cement: RelyX Ultimate	Base: Methacrylate monomers, radiopaquer, silanated fillers, initiators, stabilizers, rheological additives Catalyst: Methacrylate monomers, radiopaque, alkaline fillers, initiators, stabilizers, pigments, rheological additives, fluorescence dye, dark cure activator for Scotch bond Universal adhesive	636403/3M ESPE,
Composite resin (Filtek Z250, shade A3).	(bis-GMA, UDMA, bis-EMA, ZrO_2_/SiO_2_)	762333/3M ESPE,

**Table 2:** Shear bond strength value, Surface roughness (RA), and failure mode analysis (FA%).

Group	SBS (MPa)	Ra	FA% [A/C/M]
CA50	14.92 ± 1.43^bc^	1.69 ± 0.22^b^	[25/25/50]
CS25	12.04 ± 1.02^c^	0.36 ± 0.15^a^	[15/25/60]
B	17.97 ± 4.05^b^	4.49 ± 1.9^c^	[10/25/65]
C	22.44 ± 2.75^a^	5.2 ± 0.8^c^	[10/40/50]
D	17.55 ± 2.91^b^	4.63 ± 1.2^c^	[25/10/65]
E	18.30 ± 4.68^b^	4.93 ± 0.6^c^	[30/10/60]

Different letters within each column indicates significant difference. FA: Failure mode, A: Adhesive failure, C: Cohesive failure, and M: Mixed failure

## Data Availability

The raw data required to reproduce these findings are available upon reasonable request from the corresponding author. The processed data required to reproduce these findings are available upon reasonable request from the corresponding author.
